# Use of Heated Humidified Gases for Early Stabilization of Preterm Infants: A Meta-Analysis

**DOI:** 10.3389/fped.2018.00319

**Published:** 2018-10-25

**Authors:** Michael P. Meyer, Louise S. Owen, Arjan B. te Pas

**Affiliations:** ^1^Neonatal Unit, KidzFirst, Middlemore Hospital, Auckland, New Zealand; ^2^Department of Paediatrics, University of Auckland, Auckland, New Zealand; ^3^Department of Newborn Research, The Royal Women's Hospital, Melbourne, VIC, Australia; ^4^Murdoch Childrens Research Institute, Melbourne, VIC, Australia; ^5^Department of Obstetrics and Gynaecology, The University of Melbourne, Melbourne, VIC, Australia; ^6^Division of Neonatology, Department of Pediatrics, Leiden University Medical Center, Leiden, Netherlands

**Keywords:** preterm, hypothermia, meta-analysis, heated, humidified, gases, stabilization

## Abstract

**Background:** Large observational studies in preterm infants have shown an increase in mortality and morbidity when admission temperature is below 36.5°C. Recent randomized controlled studies have shown a reduction in admission hypothermia and an increase in the number of infants admitted with normal temperature (36.5–37.5°C) when heated humidified gases were used for initial stabilization of preterm infants.

**Objective:** The goal of this study was to perform a meta-analysis of published randomized trials using heated humidified gas compared to cold dry gas in preterm infants immediately after birth and during transport to the neonatal unit. Specific research aims were to determine the magnitude of the reduction in hypothermia and to examine neonatal outcomes including mortality.

**Methods:** A literature search was conducted in accordance with the standard methods of the Cochrane Neonatal Work Group. Randomized trials were identified and data entered into RevMan5. A fixed effects statistical model was used. Risk of bias was assessed for included studies and the GRADE approach used to determine quality of evidence. The primary outcome was admission hypothermia (< 36.5°C). Secondary outcomes included admission temperature in the normothermic range (36.5–37.5°C) and neonatal outcomes including mortality.

**Results:** Two studies met inclusion criteria and a total of 476 preterm infants were enrolled, all of whom were < 32 weeks gestation. Studies were not blinded but the overall risk of bias was low. Admission hypothermia was reduced by 36% (CI 17–50%), while admission normothermia was significantly increased. GRADE quality of evidence was high for these outcomes. The number of infants with more severe hypothermia (< 35.5°C) was significantly reduced (RR 0.32 CI 0.14-0.73). In addition, preterm infants < 28 weeks had significantly less admission hypothermia (RR 0.61 CI 0.42, 0.90) Mortality and measures of respiratory outcome were not significantly different (studies were not powered for these outcomes), though there was a trend to improvement in all respiratory measures assessed. There were no significant adverse events and no increase in admission hyperthermia (>37.5°C).

**Conclusions:** Heating and humidification of inspired gases immediately after birth and during transport to the neonatal unit improves admission temperature in preterm infants. Consideration should be given to incorporating this technique into other strategies (e.g., use of plastic wrap) designed to keep preterm infants warm on admission to the neonatal unit.

## Introduction

### Rationale

Historically, low admission temperatures in preterm infants have been associated with increased morbidity and mortality (1, 2). Recent, large cohort studies have confirmed these associations (3, 4). A Canadian Neonatal Network report on 9,833 preterm infants < 33 weeks indicated the lowest mortality with admission temperatures between 36.8 and 37.2°C and a significant increase in both mortality and morbidity if admission temperatures were below 36.5°C (5). In this study, 57% of infants had admission temperatures in the range 36.5–37.4°C. Even with use of strategies known to improve admission temperature and bundle of care initiatives to improve quality, studies have shown 25–30% of preterm infants had admission hypothermia i.e., < 36.5°C (6, 7).

In the study of (4), preterm infants < 33 weeks were more likely to be hypothermic on admission if they received positive pressure ventilation in the delivery room with unheated gas and also if they received respiratory support during transport to the neonatal unit. Each of these interventions was independently associated with admission hypothermia (OR 1.4 CI 1.03–1.88 and 1.5 CI 1.08–2.13, respectively). Respiratory support with cold dry gas is commonly used in these situations. i.e., stabilization after birth and during transport to the neonatal unit. In one study, the mean temperature of piped wall air was 23.4°C and mean relative humidity 5.4% (8). In a bench study, gases could be heated and humidified to levels recommended for patients with an artificial airway by 3 min (9). Using 50 ml water in the humidifier chamber resulted in more rapid achievement of 95% relative humidity than using larger volumes of water. It was shown that temperature and humidity of the gases fell rapidly if the power supply was discontinued (9).

Heating and humidification of inspired gases in the delivery room has been reported in an observational study (10) and more recently in randomized trials (11, 12). In all these studies there were significant improvements in admission temperatures and a reduction in admission hypothermia. In addition to the effects on admission temperature, short periods of exposure to cold dry respiratory gases have detrimental respiratory effects such as decreased lung compliance, increased work of breathing, release of proinflammatory cytokines and damage to the mucociliary layer (13–16).

The randomized studies referred to above did not result in improved outcomes, but were not powered for these outcomes. Therefore, we performed a meta-analysis to further explore these outcomes.

### Objectives

Our aim was to perform meta-analysis of existing randomized trials using heated humidified gas compared to cold dry gas for treatment of preterm infants immediately after birth and during transport to the neonatal unit.

### Specific research aims

Specific goals were to determine the magnitude of effect of using heated humidified gas in preventing admission hypothermia and to examine the effects on reported respiratory and other outcomes. Subgroup analysis for infants < 28 weeks gestation was undertaken.

## Methods

### Study design, interventions and comparators

Studies enrolling preterm infants (P) which compared use of heated humidified gas (I) with cold dry gas (C) during stabilization and transport were identified. The primary outcome of interest was hypothermia (< 36.5°C) at the time of nursery admission (O). Only randomized controlled trials were included (S).

For respiratory gases, heating and humidification (or conditioning) was said to occur if the medical gas (air or oxygen) from a piped gas supply or portable gas cylinders was passed through a heated humidification chamber. Cold dry gas was regarded as gas which was obtained directly from the piped wall supply or gas bottles and not heated or humidified.

In accordance with the World Health Organization, hypothermia was defined as an admission temperature below 36.5°C (17). Other outcomes for temperature measurements were admission normothermia (36.5–37.5°C), admission temperatures < 36 and < 35.5°C and hyperthermia (>37.5°C). Subgroup analysis of temperature outcomes for infants < 28 and < 26 weeks was undertaken. Respiratory outcomes of interest were surfactant use, intubation in the delivery room, pneumothorax, days of respiratory support and chronic lung disease (any respiratory support or oxygen at 36 weeks corrected gestation). Other neonatal outcomes to be recorded were mortality before hospital discharge, severe intraventricular hemorrhage (Grade 3 or 4), severe necrotizing enterocolitis (Bell stage 2 or more), late onset sepsis (after 48 h), retinopathy receiving laser therapy and length of hospital stay.

### Review protocol

Methods were based on the standard format of the Cochrane Neonatal [available at: neonatal.cochrane.org; (18)].Title and abstract were screened in the first instance, then full text if appropriate. We kept a record of the number of studies screened at each step. Two authors reviewed search results; differences were to be resolved by consensus or the third author if necessary. Risk of bias (low, high, or unclear) was assessed for all included trials using the Cochrane ‘Risk of bias' tool (18) The GRADE approach, (a tool to assess the strength of evidence from high to very low), and which is outlined in the GRADE Handbook (19), was used where appropriate.

### Search strategy

A comprehensive literature search was conducted including: Cochrane Central Register of Controlled Trials (CENTRAL 2017, Issue 5) in the Cochrane Library; MEDLINE via PubMed (1966 to 19 June 2017); Embase (1980 to 19 June 2017); and CINAHL (1982 to 19 June 2017) using database-specific limiters for randomized clinical trials and neonates for each database. We applied no language restrictions and used expanded MeSH terms for humidity, heat or cold and gas. Our search included references from other studies published in the medical literature, such as reviews and trials. We also searched abstracts from several conferences (Pediatric Academic Society, European Society for Pediatric Research and Perinatal Society of Australia and New Zealand).

### Data sources

We searched for randomized controlled trials, cluster-randomized, and quasi-randomized studies enrolling preterm infants (< 37 weeks' gestation).

### Data analysis

Data was entered into RevMan5v3 to calculate relative risk with 95% confidence intervals for categorical data or mean difference with 95% confidence intervals for continuous data. We used a fixed effects model.

## Results

**Figure 1:**
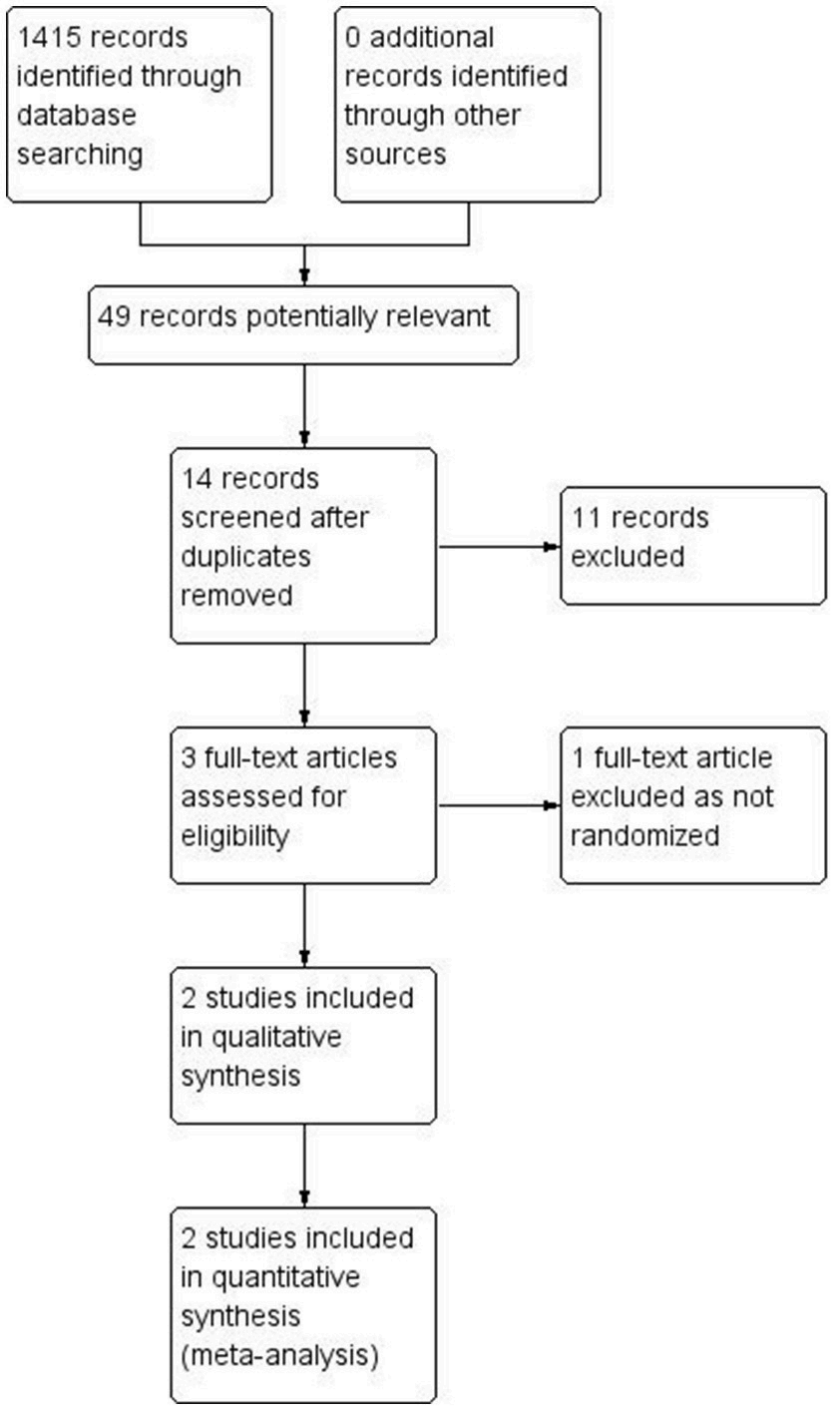
Study flow diagram.

Two studies met inclusion criteria (see Figure 1).

### Summary of identified studies

Meyer et al. (11) this randomized controlled trial was conducted at 2 sites–New Zealand and The Netherlands between 2011 and 2013. Eligible infants were < 32 weeks requiring respiratory support after cord clamping which was after 40 s in New Zealand and immediate in The Netherlands. Stratification was according to gestation and site. Heating and humidification was achieved by adding 30–50 ml water to the humidifier (MR850 Fisher & Paykel, Auckland, New Zealand) and turning on the device approximately 5 min prior to expected delivery. Infants were randomized to receive medical gas which was either heated to 37°C and humidified or cold and dry direct from the supply (wall or bottle). The median humidifier temperature (recorded in the New Zealand cohort) was 36.7°C in the humidified group. CPAP was given with a T piece resuscitator (Neopuff, Fisher & Paykel, Auckland, New Zealand). The setup of gas supply, humidifier (if used) and T piece were as described in previous studies (9, 10). Transport to the neonatal unit was carried out at one site using a radiant warmer with portable power supply and humidifier attached and abdominal temperature set to 37°C on servo control (which controls warmer output by feedback from the achieved temperature). An incubator was used at the other site. CPAP via T piece was continued until admission. The same humidifier was used for initial stabilization and transport. Admission temperature was measured in the axilla with digital thermometers and the study was powered to detect an improvement in the number of patients whose admission temperature was in the normothermic range (36.5–37.5°C).

McGrory et al. (12) this randomized controlled trial was carried out at two sites in Melbourne, Australia between 2013 and 2015. Preterm infants < 30 weeks were eligible if respiratory support was required and randomized infants were stratified by gestation and site. A humidifier (MR850 Fisher & Paykel) with 50 ml water in the chamber was used and turned on at, or just prior to, delivery. CPAP was given via T piece (Neopuff Fisher & Paykel). At one site infants were transported on the radiant warmer using the same humidification device and a portable power supply. At the other site infants were transferred to an incubator for transport and were supported using a ventilator with inbuilt heated humidification. Other details of the method (including the circuit set up) were similar to those of the study by Meyer et al. (11). The admission temperature was measured rectally and the study powered to detect a reduction in patients with admission temperature below 36.5°C.

The main differences between the studies were the cut off for gestation [ < 32 weeks in (11) and < 30 weeks in the study of (12)]. Other differences were the use of servo control in the study of Meyer et al. (11) and the use of rectal temperature in the McGrory et al. (12) study. Apart from these differences, the studies were carried out in a similar manner.

Other strategies to improve admission temperature were carried out in the two studies and these are shown in Table 1.

**Table 1:** Strategies to improve admission temperature compared between studies.

**Thermal strategy**	**Meyer et al. (11)**	**McGrory et al. (12)**
Plastic body wrap/bag	Yes	Yes
Cap/ hat	Yes	Yes
Set theater temperature(°C)	26–27	25–26
Radiant warmer for stabilization	Yes	Yes
Servo control used	Yes	No
Radiant warmer or incubator transport to neonatal unit	Yes	Yes
Power supply for transport	Yes	Yes
Heated humidified gas for transport	Yes	Yes

### Risk of bias

Risk of bias was assessed for both studies (Table 2). The two studies were generally at low risk of bias but were not blinded so there was unclear risk of performance bias and outcome assessment bias.

**Table 2:** Risk of bias table for studies of Meyer et al. (11) and McGrory et al. (12).

**Bias**	**Authors' judgement**	**Support for judgement**
Random sequence generation (selection bias)	Low risk	Computer generated
Allocation concealment (selection bias)	Low risk	Sealed envelopes
Blinding of participants and personnel (performance bias)	Unclear risk	Not blinded
Blinding of outcome assessment (detection bias)	Unclear risk	Not blinded
Incomplete outcome data (attrition bias)	Low risk	All patients accounted for
Selective reporting (reporting bias)	Low risk
Other bias	Low risk

### Data synthesis

Meta-analysis was performed on the data obtained from the two studies, which were similar in methods and design, although the McGrory et al. (12) study included infants < 30 weeks compared to infants < 32 weeks in the study of Meyer et al. (11). Collectively, 476 preterm infants were enrolled in both studies and thermal and respiratory outcomes were available for all infants.

**Figure 2:**
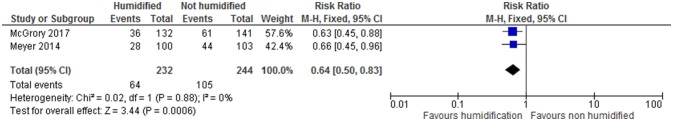
Admission temperature below 36.5°C.

**Figure 3:**
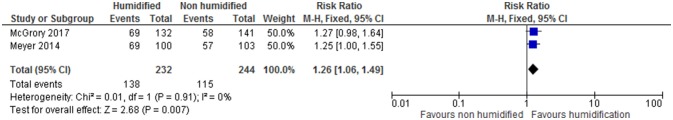
Normothermia on admission (36.5–37.5°C).

**Figure 4:**
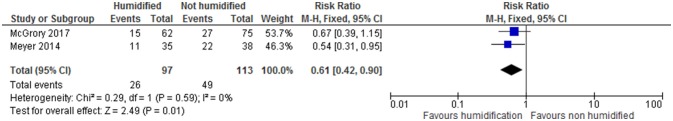
Admission temperature < 36°C.

**Figure 5:**
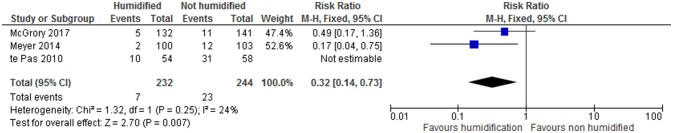
Admission temperature < 35.5°C.

**Figure 6:**
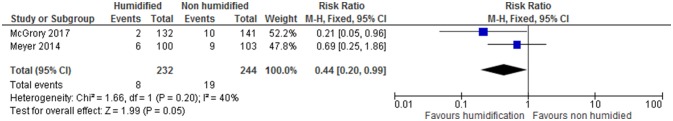
Severe intraventricular hemorrhage (Grade 3 or 4).

Days of respiratory support and hospital stay expressed as mean (SD) were obtained from the authors (unpublished). Other outcomes not reported in one or both trials (incidence of severe necrotizing enterocolitis, retinopathy requiring laser treatment, pneumothorax) were requested from the authors as well as temperature analysis in the < 26 week subgroup. Results are summarized in Table 3 (as well as the GRADE quality of the evidence) and forest plots in Figures 2–6. Other neonatal outcomes are shown in Table 4. Mean differences for admission temperature, days of respiratory support and length of hospital stay were calculated from the summary statistics presented in each trial.

**Table 3:** Heating and humidification compared with no heating and humidification of inspired gases for early stabilization of preterm infants: main outcomes and GRADE quality of evidence.

**Outcomes**	**No of participants (studies)**	**Humidification number (%)**	**Control number (%)**	**Relative effect 95% CI**	**Quality of evidence (GRADE)**	**Comments**
Admission hypothermia (< 36.5°C)	476 (2)	64/232 (27.5)	105/244 (43.0)	RR 0.64 [0.50 to 0.83]	⊕⊕⊕⊕ high	Figure 2
Admission normothermia (36.5–37.5°C)	476 (2)	138/232 (59.4)	115/244 (47.1)	RR 1.26 [1.06–1.49]	⊕⊕⊕⊕ high	Figure 3
Admission temperature (mean difference °C)	476 (2)			MD 0.16 [0.01, 0.30]	⊕⊕⊕⊖ moderate	
Admission hyperthermia (>37.5°C)	476 (2)	30/232 (12.9)	24/244 (10)	RR 1.33 [0.81, 2.17]	⊕⊕⊕⊖ moderate	
Admission hypothermia < 28 weeks	210 (2)	26/97 (26.8)	49/113 (43.3)	RR 0.61 [0.42, 0.90]	⊕⊕⊕⊖ moderate	
Admission hypothermia < 26 weeks	96 (2)	14/47 (29.7)	20/49 (40.8)	RR 0.73 [0.42, 1.27]	⊕⊕⊖⊖ low	
Severe IVH	476 (2)	8/232 (3.4)	19/244 (7.8)	RR 0.44 [0.20 to 0.99]	⊕⊕⊖⊖ low	Underpowered Figure 6
BPD or death	476 (2)	95/232 (40.9)	110/244 (45.0)	RR 0.91 [0.74 to 1.12]	⊕⊕⊖⊖ low	Underpowered
Surfactant use	476 (2)	103/232 (44.3)	123/244 (50.4)	RR 0.89 [0.74 to 1.06]	⊕⊕⊕⊖ moderate	
Intubated in delivery room	476 (2)	69/232 (29.7)	79/244 (32.3)	RR 0.92 [0.70 to 1.20]	⊕⊕⊕⊖ moderate	
Duration of respiratory support	476 (2)			RD −3.68 [−9.18, 1.82]	⊕⊕⊖⊖ low	Wide confidence intervals
Hospital stay (days)	476 (2)			MD 0.91 [−5.73 to 7.55]	⊕⊕⊖⊖ low	Wide confidence intervals

*CI, Confidence interval; RR, Risk Ratio MD, mean difference. GRADE Working Group grades of evidence: High quality: Further research is very unlikely to change our confidence in the estimate of effect. Moderate quality: Further research is likely to have an important impact on our confidence in the estimate of effect and may change the estimate. Low quality: Further research is very likely to have an important impact on our confidence in the estimate of effect and is likely to change the estimate. Very low quality: We are very uncertain about the estimate*.

**Table 4:** Other neonatal outcomes.

**Outcome**	**Number of participants (studies)**	**Humidification number (%)**	**Control number (%)**	**RR**
Death	476 (2)	20/232 (8.6)	20/244 (8.1)	1.05 [0.58, 1.90]
Chronic lung disease	476 (2)	78/232 (33.6)	92/244 (37.7)	0.90 [0.70–1.14]
Pneumothorax	476 (2)	14/232 (6)	15/244 (6.1)	0.98 [0.48, 1.99]
Necrotizing enterocolitis (stage 2 or more)	203 (1)	6/100 (6)	4/103 (4)	1.54 [0.45, 5.31]
Late onset sepsis	476 (2)	53/232 (22.8)	52/244 (21.3)	1.08 [0.77, 1.51]
Treated retinopathy	203 (1)	5/100 (5)	1/103 (1)	5.15 [0.61, 43.31]

Admission hypothermia (< 36.5°C) was reduced by 36% (CI 17–50%) from 43% in the group receiving cold dry gas to 27.5% in the heated humidified group (Figure 2) Admission normothermia (36.5–37.5°C) was significantly increased from 47.1 to 59.4% (RR1.26 CI 1.06–1.49). Mean admission temperatures were increased by 0.16°C (CI 0.01–0.3) and although the percentage of admissions with temperatures above 37.5°C increased from 10 to 12,9%, this result was not statistically significant (RR 1.33 CI 0.81–2.7). Subgroup analyses for admission hypothermia below 35.5°C (Figure 5) and for admission temperature below 36.5°C in infants < 28 weeks showed significant improvement with heated humidified gas (Table 3).

There were no significant differences in any respiratory or neonatal outcomes (Tables 3, 4), apart from a significant reduction in severe intraventricular hemorrhage (IVH) in the heated humidified group (from 7.8 to 3.4%; RR 0.44 CI 0.20–0.99).

## Discussion

### Summary of main findings and limitations of the evidence

The primary outcome, admission hypothermia (< 36.5°C), was significantly lower with relatively narrow confidence intervals in the group randomized to receive heated humidified gas during initial stabilization and transport to the neonatal unit. There was no heterogeneity in this result between the included studies (I^2^ statistic 0%). As a result of these considerations, the quality of evidence was assessed as high using the GRADE approach with future studies being relatively unlikely to change this result. The mean difference in admission temperature was, however, only slightly increased for the heated and humidified group as a whole (mean difference 0.16 CI 0.01–0.3). Nevertheless, there were significant reductions in the number of admissions with more severe hypothermia (< 36 and < 35.5°C). Fewer infants in the < 28 week subgroup had admission hypothermia with a similar trend for those < 26 weeks. Overall the heterogeneity for all assessed temperature outcomes was low. The incidence of hyperthermia was higher in the study of McGrory et al. (12). It is possible that using servo control in the early transition period could be important to avoid overheating and this requires further study. In an observational study in preterm infants < 32 weeks, admission temperatures were compared in two periods, before and after introduction of heated humidified gas for stabilization (10). Admission temperatures were significantly higher, moderate hypothermia (< 36°C) was less frequent and admission normothermia was increased in the group receiving heated humidified gas. There was no significant increase in admission hyperthermia. These results were, therefore, similar to those noted in the current meta-analysis of randomized trials.

In terms of respiratory outcomes, there were no significant differences in the outcomes assessed (intubation in delivery room, use of surfactant, pneumothorax, days of respiratory support, chronic lung disease), but there were trends to reduction in all the respiratory outcomes listed with remarkably little heterogeneity. The fact that the studies were not powered for these outcomes resulted in lower GRADE scores for quality of the evidence. There is, therefore, at least a moderate likelihood that results will change if further studies are done and future studies should continue to report these outcomes.

There was a significant reduction in severe IVH in the group receiving heated humidified gas. However, numbers were small with wide confidence intervals and the quality of evidence as judged by GRADE score was low. Whilst preterm infants may be at risk of both hypothermia and severe IVH, there was no evidence that infants in the control group with severe IVH had lower admission temperatures (unpublished data from both studies). Overall, the observed lower rate of severe IVH may be a chance finding.

Mortality rates were very similar in the control and intervention groups in spite of the observed increase in admission temperature. However, the studies were not powered for this outcome. Data from the Canadian Neonatal Network (5) showed an increase in mortality from 5.2 to 8.2% when admission temperature fell below 36.5°C (RR 1.63 CI 1.39–1.93). To observe a similar decrease in mortality in randomized trials would require almost 1,100 infants per group (alpha 0.05, beta 0.8). The mortality result from the randomized trials, therefore, has a very low GRADE score. Other neonatal outcomes were not significantly different.

Whilst no adverse effects of using heated humidified gas were reported in the studies, care needs to be taken to avoid hyperthermia. In addition, the respiratory circuit set up adds a level of complexity during a potentially stressful time. This could be mitigated by early preparation and checking for leaks by ensuring adequate pressure is generated when the T piece resuscitator is in use. It appears that the humidifier can be turned on at, or shortly before delivery with 30–50 ml of water in the chamber, confirming the results of bench top studies (9, 20). If the humidification chamber is filled, it takes longer to fully warm up (21) but does start to warm the gases as soon as it is turned on. The extra cost of the circuits also needs to be considered. At the time of the studies this was approximately US$50 per infant (a different circuit is required for respiratory support once the T piece is no longer being used).

This meta-analysis strengthens the evidence favoring improvement in admission temperature in infants < 32 weeks with the early application of heat and humidity during delivery room stabilization and transport. This may be particularly relevant for infants < 28 weeks gestation. To determine whether there are other benefits (particularly respiratory), much larger studies will need to be done. Whilst it is important to do these, further information on less common neonatal outcomes could be provided by historic observational studies.

## Conclusions

We recommend considering heating and humidification of inspired gases during stabilization after birth and transport to the nursery as one of the measures to improve admission temperatures in preterm infants.

## Author contributions

All three authors conceived the study and were involved in the literature search. MM carried out the data entry and wrote the first draft. LO and AtP checked data entry and provided critical input for the manuscript.

### Conflict of interest statement

The authors declare that the research was conducted in the absence of any commercial or financial relationships that could be construed as a potential conflict of interest.
